# Advanced tensile testing as a new tool to quantify properties of food

**DOI:** 10.1016/j.crfs.2023.100577

**Published:** 2023-08-30

**Authors:** Miek Schlangen, Erik Schlangen, Atze Jan van der Goot

**Affiliations:** a Laboratory of Food Process Engineering, Wageningen University, PO Box 17, 6700 AA, Wageningen, the Netherlands; b Microlab, Faculty of Civil Engineering and Geosciences, Delft University of Technology, Delft, the Netherlands

**Keywords:** Tensile test, Meat analogue, Mechanical property, Poisson's ratio, Digital image correlation

## Abstract

Mechanical properties of food products are regularly analysed by tensile tests. The aim of this study was to demonstrate the potential of using advanced tensile testing techniques to better understand the mechanical properties of anisotropic food products, such as meat analogues and certain dairy products. The effects of various tensile testing parameters, including tensile gauge length and deformation rate, on the interpretation of mechanical properties of meat analogues was studied. Additionally, digital image correlation, an image analysis technique, was used for true distance recording and analysis of fracturing behaviour of the products. An isotropic product was prepared from solely soy protein isolate, and an anisotropic product was prepared from soy protein isolate and pectin using the shear cell technology. The tensile properties of the products were studied with four different moulds with varying gauge lengths of 17.5, 15, 11.5, and 8.5 mm, and at three deformation rates of 46.2, 23.1, and 11.6 mm/min. A smaller gauge length and slower deformation rate improved visualization and interpretation of the multi-stage descending branch in force – distance curves of anisotropic products. Additionally, tensile parameters, specifically toughness, proved to be more accurate at small gauge length and slow deformation rate, because overestimation due to rapid crack propagation was prevented. True distance data obtained with digital image correlation further improved the interpretation of the fracturing behaviour of the products. Inhomogeneous strain distribution in anisotropic products was shown with digital image correlation, in contrast to the homogeneous strain distribution observed in isotropic products. Furthermore, the Poisson's ratio, obtained through digital image correlation, explained inherent differences in structure and plasticity between isotropic and anisotropic meat analogues. This study shows the importance of careful selection of testing parameters and techniques. Moreover, it advises the use of digital image correlation for better measurement of fracture mechanics and strain distribution.

## Highlights

• Tensile gauge length and deformation rate were optimized for data interpretation.
• Digital image correlation was used to obtain true distance data and Poisson's ratio.
• Anisotropic products exhibited heterogeneous, multi-stage fracture patterns.
• Isotropic products exhibited homogeneous fracture patterns.

## Graphical abstract

**Figure:**
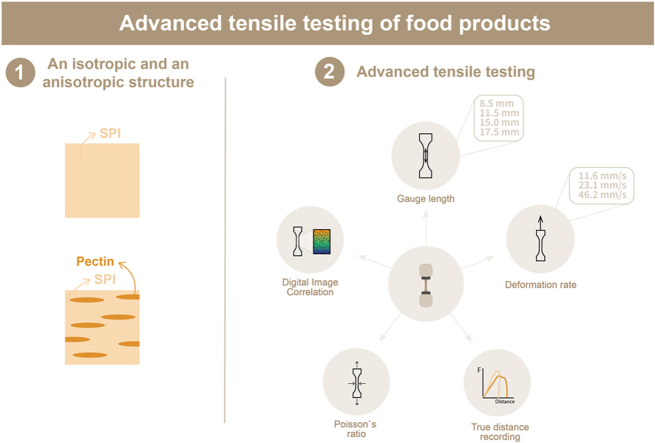

## 1 Introduction

Preparation and consumption of foods normally involve large deformations. The mechanical and fracturing behaviour of foods becomes complex at large deformations, but at the same time important to understand in product development (Lu and Abbott, 2004). The mechanical and fracturing behaviour can be studied using various mechanical tests, such as compression tests, Warner Bratzler tests, and tensile tests (Schreuders, Schlangen, Kyriakopoulou, Boom and van der Goot, 2021). Among the different methods, tensile tests are commonly used to characterize anisotropic structures of food products, such as certain dairy products and meat analogues (Ak et al., 1993; Ak and Gunasekaran, 1997; Dekkers, Nikiforidis and van der Goot, 2016).

A tensile test is a mechanical analysis to study the resistance of a material against tearing. In a tensile test, a material is placed between two grips and extended in a uniaxial direction at a fixed strain rate. When a force is applied to a food product, it will fracture at the weakest points, which are often located at the interfaces between dispersed and continuous phases. Comprehending the fracture behaviour of food products can provide insights into their multi-phase structure, which makes it possible to tailor the fracture characteristics as desired. Previously, it has been shown that tensile testing is regularly used to evaluate the texture of meat and meat analogue structures as well as dairy products, such as mozzarella (Ak and Gunasekaran, 1997; Schreuders et al., 2021). Currently, most studies on food that use tensile tests report a few tensile parameters: fracture stress, fracture strain, and Young's Modulus (Chen et al., 2022; Mcclements et al., 2021; Pietsch et al., 2019; Schreuders et al., 2019). Here, the fracture stress is defined as the maximum stress before a rapid decrease in stress, the fracture strain is defined as the strain at the fracture stress, and the Young's Modulus is defined as the slope of the elastic part of the stress-strain curve. However, the potential of tensile tests for food products is not yet fully utilized. There is little to no effort in literature on finding the exact tensile testing parameters to use and how to obtain any additional tensile parameters such as Poisson's ratio and local strain.

**Fig. 1:**
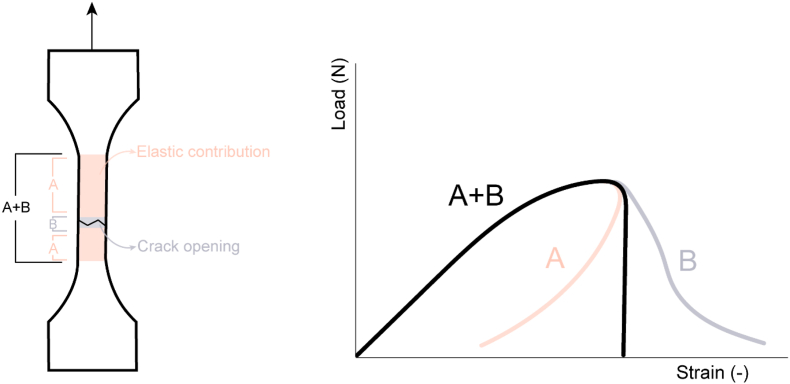
Schematic illustration of the two contributions to the force – strain results: elastic contribution and crack opening.

We, therefore, analysed how the method is applied to non-food materials following material science rules. It has been established in related disciplines that the rate of tensile testing and the tensile gauge length can have a significant impact on test outcomes (Ak and Gunasekaran, 1997; Bate et al., 2008; Hertsberg, 1996). The force – strain results of a tensile test always consist of two contributions: (1) the crack opening and (2) the elastic deformation over the total gauge length (Fig. 1) (Hordijk, 1991). While the crack opening remains constant, the elastic deformation changes when the gauge length is altered. When the gauge length is too long, the elastic contribution is large and will lead to unstable/undetailed fracture. An accurate measurement of fracture energies and analysis of the force-deformation curve requires a stable displacement-controlled experiment. Certain materials, such as meat analogues, are characterized by relatively high crack velocities and often exhibit brittle behaviour. To mitigate this issue, it is essential to minimize the elastic energy in the specimen. One possible approach is to reduce the size of the specimen (van Mier, 1997). Thus, a proper selection of the testing rate and gauge length will allow greater insights from the resulting data. These insights can, then, be employed to gain a comprehensive understanding of how to enhance meat analogue structures.

Furthermore, conventional tensile testing uses linear variable differential transformers (LVDT's) to precisely measure the deformation of (part of) the samples. However, the application of LVDT's on soft samples is complex, and often unprecise. A virtual extensometer via, for example, digital image correlation (DIC) is an alternative for LVDT's. Previously, it was proven that DIC correlated excellently with LVDT measurements in human tendons (Luyckx et al., 2014). However, the application of DIC for food materials is unexplored, except for one study on surimi gels (Park et al., 2023).

Therefore, the objective of this study is to show how testing conditions impact tensile testing results and to demonstrate the potential of DIC to facilitate better interpretation of mechanical properties of food products. Those findings will be presented as both raw and processed tensile data. It is important to mention that anisotropic structures of food products can be evaluated at different length scales. This study focusses on macroscopic properties of food products, but we acknowledge that structural properties over multiple length scales are important. Here, the extensive mechanical properties of two distinct structures were studied: a homogeneous, isotropic product composed of soy protein isolate, and a heterogenous, anisotropic product composed of soy protein isolate and pectin. The latter product has to be described as a water-in-water emulsion of pectin in soy protein isolate, and can be readily transformed into an anisotropic and often fibrous product through shear structuring. However, the chosen materials in this study are solely model products to demonstrate the potential of advanced tensile testing to get better insights in the mechanical properties of isotropic and anisotropic food products.

## 2 Materials & methods

### 2.1 Materials

Soy protein isolate (SPI) (Supro 500 E IP) was obtained from Solae (DuPont, St. Louis, MO, USA). SPI had a protein content of 81.7 ± 1.1% based on dry weight (N x 5.7 as measured by Dumas). The dry matter content of SPI was 93.7 ± 0.2%. Pectin from citrus peel (SLBQ6929V) (high methylated, 92.2 wt% dry matter) was obtained from Sigma-Aldrich (Zwijndrecht, the Netherlands). White paint and black spray paint were obtained from Flexa (AkzoNobel, Sassenheim, the Netherlands) and OK (European Aerosols, Wolvega, the Netherlands), respectively.

### 2.2 Methods

#### 2.2.1 Preparation of SPI and SPI-pectin blends

Two biopolymer mixtures (SPI and SPI-pectin) were prepared with a constant dry matter content of 44 wt%. Previous research found that SPI alone forms an isotropic structure, while SPI combined with pectin forms an anisotropic structure (Dekkers et al., 2016). The addition of pectin was thus specifically chosen for its positive impact on the texture. The SPI blend comprised of 44 wt% SPI (dry based) and 56 wt% demi water. The SPI/pectin blend comprised of 41.8 wt% SPI (dry based), 2.2 wt% pectin (dry based) and 56 wt% demi water. First, the SPI was mixed with the demi water with a spatula and left to hydrate for 30 min. Then, in the case of the SPI-pectin blend, pectin was added to the hydrated sample and mixed thoroughly.

#### 2.2.2 Shear-induced structuring with the high temperature shear cell

**Fig. 2:**
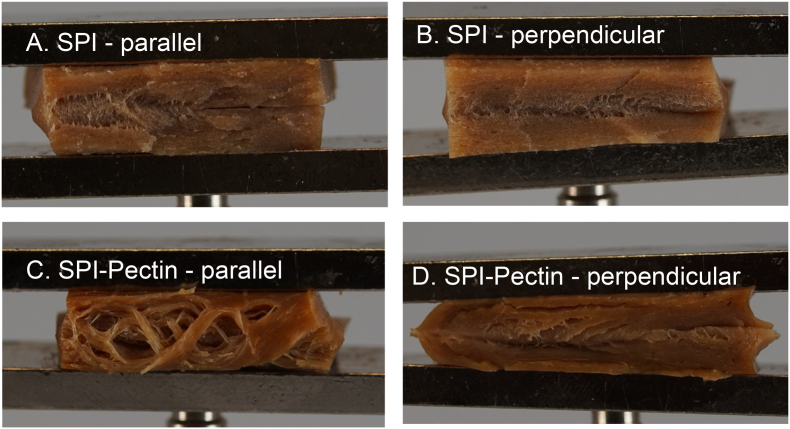
Representative macrostructures of meat analogues with A. SPI in parallel direction to shear flow, B. SPI in perpendicular direction to shear flow, C. SPI-Pectin in parallel direction to shear flow, and D. SPI-Pectin in perpendicular direction to shear flow. Samples are approximately 1.5 cm in width.

The prepared blends were structured into products with the high temperature shear cell (HTSC). The method for shear-induced structuring with the HTSC was based on previous research by Dekkers, Nikiforidis and van der Goot (2016). SPI and SPI-pectin blends were placed into the pre-heated HTSC and not sheared at 0 s^−1^ for SPI and sheared at 39 s^−1^ for SPI/pectin for 15 min at 140 °C. Subsequently, the HTSC was cooled down to 25 °C within 5 min, after which the samples were taken out and placed in plastic bags to prevent moisture loss. The samples were frozen at −18 °C before further analysis. HTSC samples were prepared in triplicate. The macrostructures of the isotropic SPI and anisotropic SPI-Pectin products are shown in Fig. 2.

#### 2.2.3 Tensile strength analysis

**Fig. 3:**
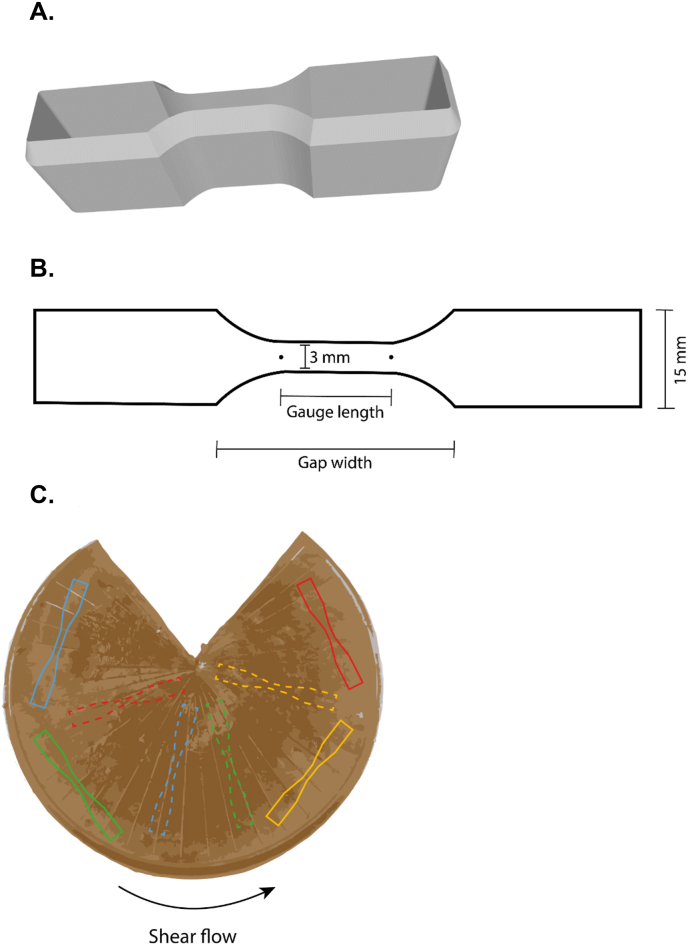
Visual representation of A. 3D model of tensile mould, B. tensile mould dimensions, C. position of specimen cut from meat analogue with full lines parallel to shear flow direction and dotted lines perpendicular to shear flow direction. Gauge lengths were 8.5 mm (red), 11.5 mm (blue), 15 mm (yellow), 17.5 mm (green). (For interpretation of the references to colour in this figure legend, the reader is referred to the Web version of this article.)

Tensile strength analysis of the HTSC products was performed using a Texture Analyser (TA.XTPlusC, Stable Micro Systems, Surrey, United Kingdom) equipped with a 5 kg load cell. An incision was made in the HTSC product so that it could lay flat. Specimens were cut from the HTSC products in parallel and perpendicular direction to the shear flow with dog-bone-shaped moulds (Fig. 3B and C). The dog-bone-shape was specifically chosen to direct the stress to the middle of the specimen for controlled fracture. These moulds were 3D printed in four different sizes (Table 1) (Fig. 3A) to study the effect of the gauge length on tensile parameter extraction. Moulds were designed and printed with the intention of incorporating a sharp diagonal edge, thereby ensuring optimal cutting performance (Fig. 3A). Specimens were given a dog-bone shape to ensure that crack propagation and eventually fracturing occurred in the measurement zone. The thickness and width of the cut specimen varied (due to the shape of the cone-in-cone HTSC) and were, thus, measured and accounted for in calculation of stress and strain. The ends of the specimens were placed into the sandpaper-coated tensile grips with a gap width of 20, 23.3, 26 or 30.5 mm dependent on gauge length (Table 1).

**Table 1:** Gauge length and gap width of 3D printed tensile moulds, and corresponding deformation rate.

	Gauge length (mm)	Gap width (mm)	Deformation rate (mm/min)
Mould 1	17.5	30.5	70.4
Mould 2	15.0	26.0	60.0
Mould 3	11.5	23.3	53.8
Mould 4	8.5	20.0	46.2, 24.1, and 11.5

Before implementing the tensile test, the frontal surface of the specimens was painted with a thin layer of white acrylic paint. Afterwards, black spray paint was used to create randomly distributed black dots on the white specimen for employing digital image correlation (DIC). A uniaxial tensile test was performed at room temperature with a deformation rate of 46.2, 53.8, 60 or 70.4 mm/min dependent on gauge length (Table 1). The smallest mould (8.5 mm gauge length) was also analysed at 24.1 and 11.5 mm/min. The force and displacement were recorded by the Exponent Connect Software (Stable Micro Systems, Surrey, United Kingdom). The true stress σ (Pa) was calculated with equation (1):

(Eq. 1) $$\sigma \left(t\right)=\frac{F\left(t\right)}{A\left(t\right)}\;\left[Pa\right]$$

where *F(t)* is the force per unit of area *A(t)*. Here, we assume that the specimen's volume did not change during measurement, i.e. that the material's Poisson ratio (transverse strain/axial strain) is equal to 0.5. The area *A(t)* is a dynamic value and is calculated with equation (2):

(Eq. 2) $$A\left(t\right)=\frac{h_{0}}{h\left(t\right)}\times A_{0}\;\left[m^{2}\right]$$

where *h*_*0*_ is the initial gauge length (m) at t = 0, *h(t)* is the gauge length at time t, *A(t)* is the cross-sectional area A (m^2^) of the tensile bar at time t, and *A*_*0*_ is the initial cross-sectional of the specimen calculated by multiplying the thickness and width of the specimen.

The true strain ε (−) was calculated with equation (3):

(Eq. 3) $$\varepsilon_{h}\left(t\right)=\ln\frac{h\left(t\right)}{h_{0}}$$

The fracture point was defined as the point following a sharp decrease in stress in the stress-strain curve. The Young's Modulus, or stiffness, was defined as the slope of the linear part of the stress-strain curve. The toughness was defined as the area under the stress-strain curve. The anisotropy indices were calculated with equation (4).

(Eq. 4) $$Anisotropy\;index=\frac{Tensile\;parameter\;parallel\;to\;shear\;direction}{Tensile\;parameter\;perpendicular\;to\;shear\;direction}$$

#### 2.2.4 Digital image correlation

**Fig. 4:**
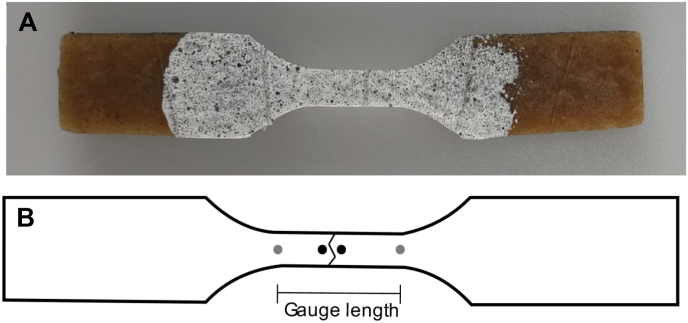
A) Example of painted specimen for tensile testing and DIC analysis and B) example of virtual extensometer points with grey points indicating the gauge length and black points indicating the area around the crack. The vertical lines in A) are an intrinsic property of the HTSC product due to grooves on the surface of the cones to prevent slip. These lines did not affect the measurements as far as the authors are aware.

Digital Image Correlation (DIC) was utilized during tensile testing to obtain true deformation values and to further understand the fracture mechanics of the specimen. Prior to tensile testing, the frontal surface of the specimen was painted white (Wolkenwit Kleurtester, Flexa, AkzoNobel Decorative Coatings BC, Sassenheim, the Netherlands) and randomly distributed black dots were applied with black spray paint (OK Spuitlak Mat, European Aerosol s, Wolvega, the Netherlands) (Fig. 4A). This pattern is necessary to enhance the contrast for DIC analysis. A Sony 4 K FDR-AX53 equipped with a Zeiss 2.0/4.4–88 mm lens was utilized to acquire a video at 25 fps during the test process. The open source Ncorr2 and Ncorr_Post software were employed for DIC analysis (Blaber et al., 2015; Nežerka et al., 2016). All frames of the video were loaded into the Ncorr2 software and analysed. The region of interest was specified manually and the software formed a virtual grid with subsets. The deformation of each subset, a set of pixels, was monitored based on the reference image. The reference image was the image taken before deformation was applied. The subset position of the reference image was described as P (x,y), while the position of the deformed image is described as P′ (x’,y’). Monitoring of all subset movements occurred through correlation. Subsequently, the DIC results were loaded into Ncorr_Post to obtain displacement data. Here, a virtual extensometer was utilized to measure the displacement between two distinct sets of points on the reference image. The first two points were selected as the limits of the gauge section, while the second two points were selected in the vicinity of the crack area with a distance of 3 mm (Fig. 4B). The true distance data for each analysed frame was synchronized with the corresponding time and force measurements recorded by the texture analyser. Due to the high computational intensity of DIC analysis, only one specimen per condition was analysed.

## 3 Results & discussion

### 3.1 Effect of gauge length

**Fig. 5:**
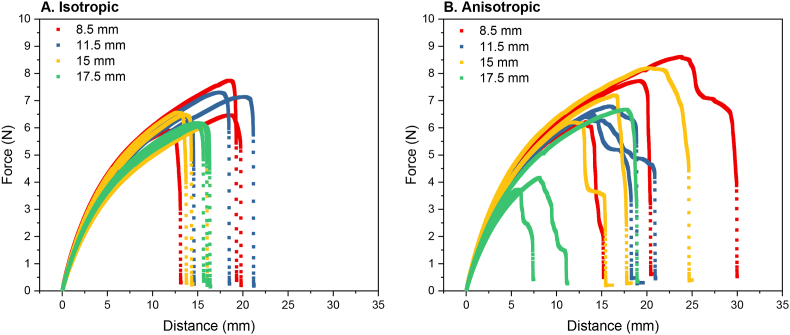
Force-distance curves of A: Isotropic and B: Anisotropic products with varying gauge lengths measured parallel to the shearing direction. Red = 8.5 mm, blue = 11.5 mm, yellow = 15 mm, 17.5 mm = green. Measurements were performed in triplicate and individual curves are shown. (For interpretation of the references to colour in this figure legend, the reader is referred to the Web version of this article.)

**Fig. 6:**
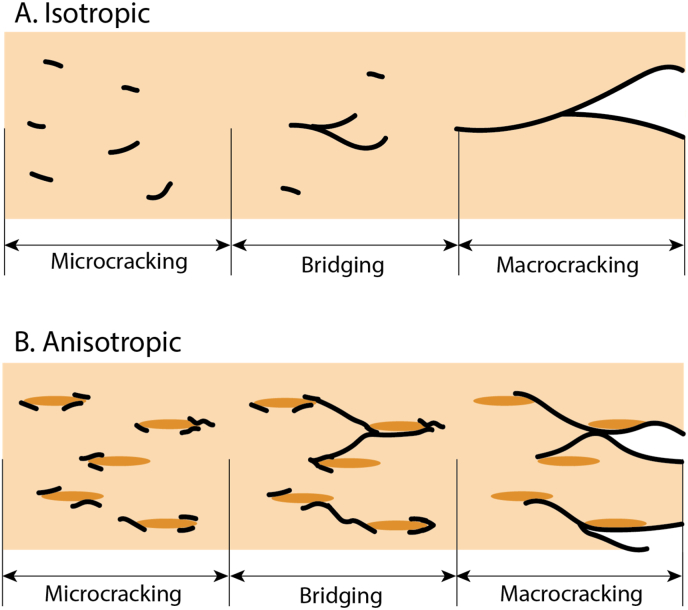
Mechanisms of fracturing in A) homogeneous isotropic products and B) heterogeneous anisotropic products. Black lines indicate cracks.

The effect of gauge length was evaluated by interpreting the force-distance curves of isotropic and anisotropic products measured parallel to the shearing direction (Fig. 5). Here, the results are presented as individual curves rather than averages. The variation between the individual curves can be seen as a measure for heterogeneity in structure, which is a result of the anisotropy of the product. Gauge length affected the outcomes of the measurements of anisotropic products more than isotropic products. In isotropic specimen, we observed that force increased with distance until a fracture point, which was evidenced by a rapid decrease in force (Fig. 5A). In general, homogeneous materials tend to behave more brittle (van Mier, 1997). This agrees with the results of our isotropic product consisting of SPI, which is considered as a relatively homogeneous material. The curve shapes between different gauge lengths were similar in the isotropic specimen (Fig. 5A). Furthermore, variance between triplicates was small for the 15 mm and 17.5 mm gauge length, while it was slightly larger for the 11.5 and 8.5 mm gauge lengths. The 8.5 and 11.5 mm gauge lengths also reached a larger distance and higher force at fracture (Fig. 5A). While this was unexpected, we believe that this may be due to the extension outside the gauge section of the specimen. The contribution of the extension outside of the gauge section was larger in short specimen compared to large specimen. Additionally, there is the chance that specimen with longer gauge sections contain more defects or inhomogeneities than the specimen with shorter gauge lengths (Liu et al., 2017). Therefore, the longer specimen were more likely to suffer from fracture earlier in the tensile test procedure. In anisotropic specimen, the variance between triplicates was rather large, indicative of a heterogeneous material (Fig. 5B). The tensile curve shape of anisotropic specimen was also dependent on gauge length. For a gauge length of 15 mm, we observed that the force increased with distance, and rapidly decreased after the fracture point. For smaller gauge lengths (11.5 and 8.5 mm), the material fractured in multiple stages. The fracturing process is then considered to be a zone of discontinuous microcracking ahead of a continuous macrocrack (van Mier, 1997). A long fracture process was expected for the anisotropic products, where inhomogeneities lead to both weaker and stronger areas in the sample. In tensile testing, the nucleation of microcracks is the first phase of the fracture process (Fig. 6). Then, bridging of these microcracks occurs, which causes automatic propagation to a macrocrack (Fig. 6A). In isotropic products, the propagation into a macrocrack proceeds quickly, as evidenced by the rapid decrease in stress at the fracture point (Fig. 5A). However, the bridging of microcracks can be inhibited by the presence of a dispersed phase, such as pectin. The dispersed phase can delay the onset of macrocracking or fracturing (Fig. 6B). This phenomenon is reflected in the distinctive shape of the descending branch observed in the force-distance curve of the anisotropic specimen, which exhibited a prolonged decline compared to the isotropic specimen (Fig. 5B). The contribution of the elastic deformation was smaller for shorter gauge lengths. Thus, the use of shorter gauge lengths provided a better approximation of the true fracturing behaviour of the products compared to longer gauge lengths (van Mier, 1997).

It is important to take two considerations into account when deciding on the gauge length. First, the gauge length must be larger than the defects inside the material that will control the fracture (Luyten et al., 1992). Defects of approximately 750 μm were found in previous research on anisotropic meat analogues (Dekkers et al., 2016). Thus, this sets a lower limit of gauge length. Second, the gauge length must be small enough to get continuously increasing deformation (van Mier, 1997). Here, gauge lengths of 8.5 mm were chosen for continuation of further experiments based on the considerations described above.

### 3.2 Effect of texture analyser deformation rate

**Fig. 7:**
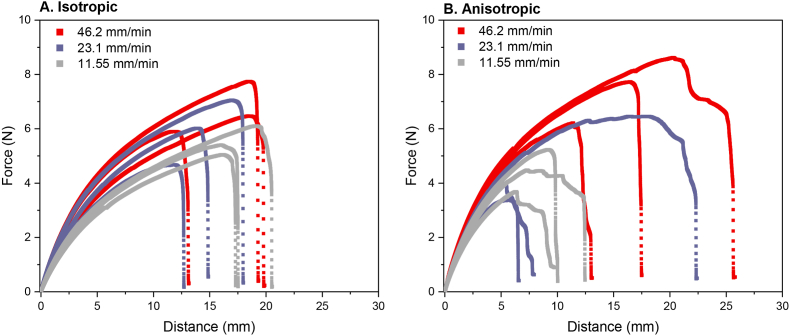
Force-deformation curves of A: Isotropic and B: Anisotropic products with gauge length 8.5 mm at varying deformation rate. Red = 46.2 mm/min, purple = 23.1 mm/min, grey = 11.55 mm/min. Measurements were performed in triplicate and individual curves are shown. (For interpretation of the references to colour in this figure legend, the reader is referred to the Web version of this article.)

The effect of three different deformation rates (46.2, 23.1, and 11.55 mm/min) was evaluated by interpreting the force – distance curves in isotropic and anisotropic products with a gauge length of 8.5 mm (Fig. 7). Here, we observed that deformation rate had a different effect on the isotropic products than on the anisotropic products. In isotropic specimen, decreasing the deformation rate led to a more defined and rounded off fracture point (Fig. 7A). In anisotropic specimen, decreasing the deformation rate led to a shorter fracture distance (Fig. 7B). Furthermore, the fracture propagation was slower at 11.55 mm/min, as evidenced by a more defined fracture zone. Propagation of microcracks into macrocracks probably proceeded slower at a lower deformation rate, which is beneficial for understanding the fracture properties (Fig. 6). The energy storage and dissipation mechanisms in viscoelastic materials are reliant on their relaxation times. Consequently, the measurements of the material properties are impacted by the rate of deformation (Ferry, 1980). This was for example evident in the isotropic specimen where a deformation rate of 11.55 mm/min induced a degree of relaxation in the product, as evidenced by a more concave curve shape (Fig. 7A). This was not observed at the higher deformation rate of 46.2 mm/min (Fig. 7A). Lower deformation rates provided more time for dissipating mechanisms to transpire, which led to lower stiffness. This relationship between deformation and material properties has been previously observed by Ak et al. (1993), Schab et al. (2022), and Sliwinski et al. (2004) in tensile testing of mozzarella cheeses, caramel and wheat dough, where lower deformation rates resulted in decreased fracture stresses.

The observations described above suggest that the fracture process is composed of multiple stages. This indicates the heterogeneity of the product, which leads to weaker areas that fracture initially, and relatively stronger areas that fracture later. We can thus argue that increased anisotropy or heterogeneity of the product leads to a more extended descending branch. In the anisotropic specimen it was also clear that deformation rate did not change the linear part of the curve, but only affected the non-linear and fracture zone of the curve (Fig. 7B). Specifically for anisotropic specimen, a slower deformation rate was, thus, beneficial for interpreting the tensile test results. Therefore, a gauge length of 8.5 mm and a deformation rate of 11.55 mm/min were used in the following results.

### 3.3 Tensile parameters

**Fig. 8:**
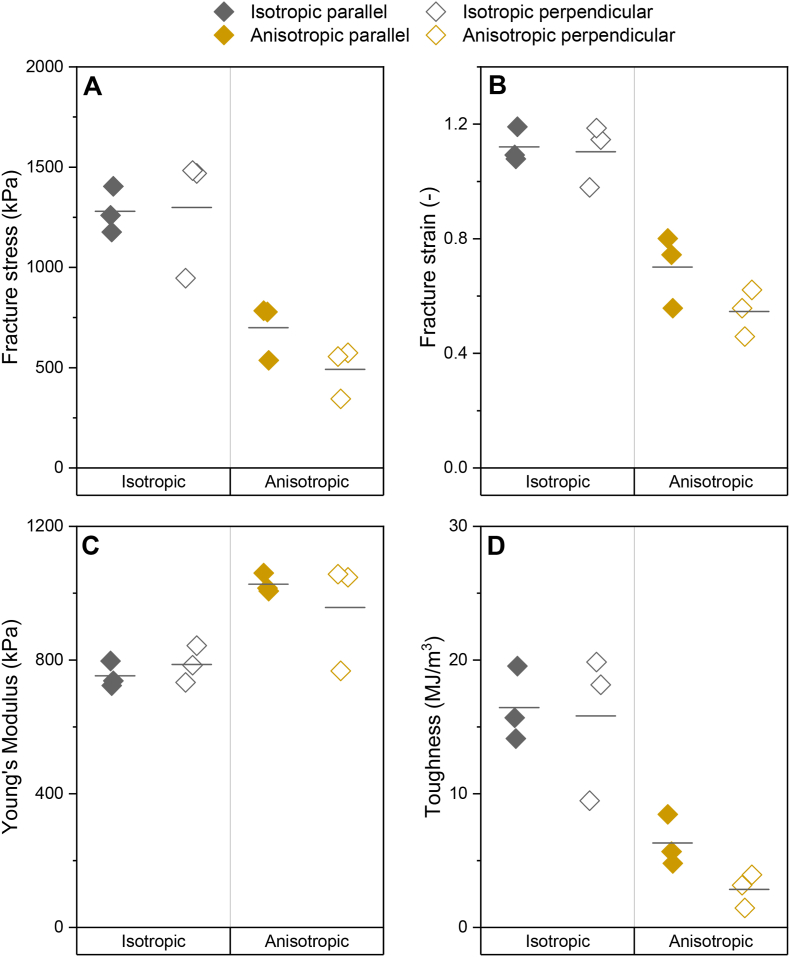
A) Fracture stress, B) Fracture strain, C) Young's Modulus, D) Toughness of an isotropic and an anisotropic product analysed with a gauge length of 8.5 mm and a deformation rate of 11.55 mm/min. Specimen were analysed in the direction parallel (closed symbols) and perpendicular (open symbols) to the shear flow. Tests were performed in triplicate and individual data points are shown. Horizontal line indicates mean value.

**Fig. 9:**
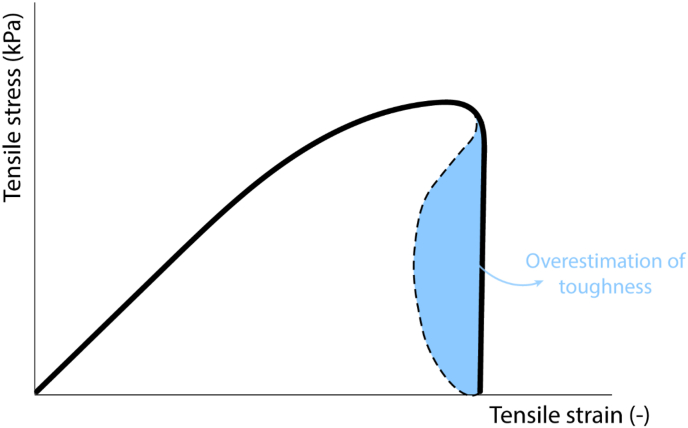
Schematic illustration of overestimation of the toughness in case of isotropic products.

Various tensile parameters exist that can be extracted from the stress – strain curves. Here, we will describe fracture stress, fracture strain, Young's Modulus, and toughness for an isotropic product and an anisotropic product (Fig. 8). There were clear differences in tensile parameters between the isotropic and anisotropic product. We found that anisotropic specimen had a lower fracture stress, fracture strain, and toughness compared to the more homogeneous, isotropic specimen (Fig. 8A, B, D). However, anisotropic specimen did have a higher Young's Modulus than isotropic specimen (Fig. 8C). Thus, anisotropic specimen were weaker, yet stiffer, through the addition of a dispersed pectin phase to SPI to make the product anisotropic. Products with a high toughness, such as the isotropic specimen in this study, are considered to be ductile materials, while products with a low toughness, such as the anisotropic specimen in this study, are considered to be more brittle (Hertsberg, 1996; Schab et al., 2022). However, the toughness results of the isotropic products have to be interpreted with caution. The absolute values of the toughness are probably overestimated in the case of (especially) the isotropic product, due to the static, continuous deformation rate (Fig. 9). If available, it would be better to use dynamic strain control in tensile testing to overcome this issue. In dynamic strain control, the controlling factor in tensile testing is the strain rather than the displacement, resulting in enhanced stability of the crack propagation (van Mier and van Vliet, 2002).

**Fig. 10:**
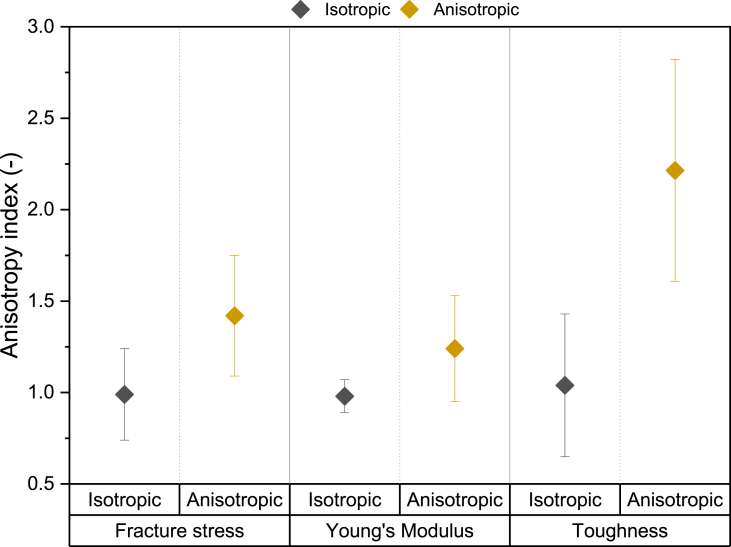
Anisotropy index of fracture stress, Young's Modulus, and toughness of isotropic and anisotropic products analysed with a gauge length of 8.5 mm and a deformation rate of 11.55 mm/min. Specimen analysed in the direction parallel to shear flow were divided by specimen analysed perpendicular to shear flow. Tests were performed in triplicate and mean values including standard deviation are shown.

The anisotropy index of the fracture stress is often found to correlate with fibrousness in case of soy-based products (Dekkers et al., 2016; Schreuders et al., 2019; Schreuders et al., 2021). Here, we observed that the anisotropy index of the fracture stress, Young's Modulus, and toughness of isotropic products was approximately 1 (Fig. 10). This was as expected, because the isotropic products had not been sheared. Indeed, the fracture stress anisotropy index of the anisotropic products was higher than 1, confirming the anisotropic nature of the products (Fig. 10). Previous research by Dekkers et al. (2016) also showed an anisotropy index higher than 1 upon addition of pectin to SPI and attributed this to weakening of the structure due to the orientation of the dispersed pectin phase in the perpendicular direction. They suggested that the dispersed phase either forms weak areas in the composite, or the adhesion between the dispersed and the continuous phase is weak. We introduce the anisotropy index of the Young's Modulus and toughness as new tensile parameters for meat analogue analysis. The Young's Modulus anisotropy index of the anisotropic products was only slightly higher than 1. This was expected, because large deformation properties, such as fracture stress, are much more affected by weak areas in the sample, such as pectin, than small deformation properties (van Vliet, 1996). The occurrence of anisotropy in the linear region of the Young's Modulus is, therefore, unlikely. A previous study, however, did show the importance of a high anisotropy index (∼7.3) of the Young's Modulus in cooked chicken (Schreuders et al., 2019). Quantifying anisotropy in the linear regime of the stress-strain curve is therefore still an important parameter to describe similarities and differences between meat and meat analogues. At low strains, the stress-strain curves for both parallel and perpendicular specimen show similar behaviour because primary deformation is taking place in the continuous phase gel network. However, as the strain exceeds the linear region, the perpendicular specimen exhibited lower strain values at fracture (Fig. 8). This was likely due to the presence of relatively weak dispersed-continuous phase interfaces in this direction, as previously described by Dekkers et al. (2016). Conversely, the fracture of the anisotropic specimen in the parallel direction probably occurred within the continuous phase, since there were fewer weak interfaces in this direction. Fibrousness would thus be better detected by fracture parameters rather than Young's Modulus. A fracture parameter is, amongst others, the toughness. The toughness anisotropy index of anisotropic specimen was higher than 2, further confirming the anisotropic nature of these products (Fig. 10). This is striking because the absolute toughness in the parallel direction of the anisotropic specimen was rather low (Fig. 8D). These results suggest that the anisotropy index of toughness may be a more effective metric for distinguishing variations in properties between isotropic and anisotropic products compared to the anisotropy index of fracture stress and Young's Modulus. This would become even more evident once the overestimation of the toughness of the isotropic product would be reduced.

### 3.4 Poisson's ratio and true distance recording with DIC

**Fig. 11:**
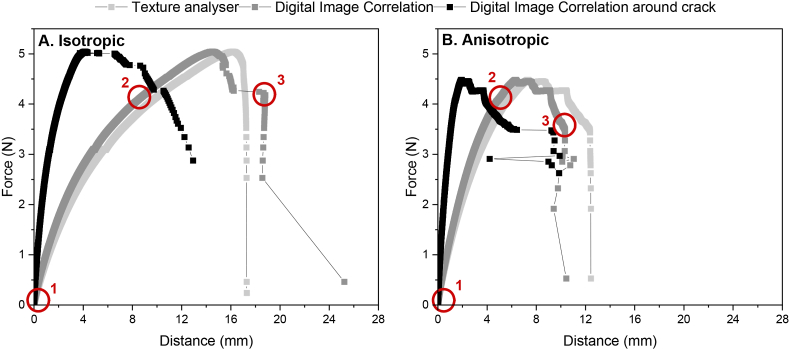
Force – distance curves recorded by texture analyser (light colour), digital image correlation of the gauge length (medium colour), and digital image correlation around the crack (dark colour) for A. Isotropic and B. Anisotropic products. A gauge length of 8.5 mm was used for all specimen. Circles with numbers correspond to DIC images in Fig. 14, with 1: start, 2: halfway, and 3: crack. (For interpretation of the references to colour in this figure legend, the reader is referred to the Web version of this article.)

In mechanical analysis studies that involve tensile testing, the deformation rate recorded by the texture analyser is generally used. However, in most cases, this leads to overestimations of certain tensile parameters. Furthermore, the deformation of the tensile grips and load cell contribute to the recorded distance, and are thus influencing the results. Therefore, we studied the difference between using the distance recorded by the texture analyser and the true distance from digital image correlation (DIC) (Fig. 11). Here, the distance recorded by the texture analyser takes into account the complete gap section between the tensile grips, while the distance recorded by DIC is determined over specified virtual extensometer points in the image, being the gauge section and the area around the crack (3 mm) (Fig. 4B). For both isotropic and anisotropic specimen, we observed differences in distance recorded by the texture analyser compared to DIC (Fig. 11A and B). The force-distance curves obtained from the texture analyser and DIC were expected to be similar in terms of the overall trend of the graph, as both methods measure the same physical quantities. However, there may be differences in the details of the curves. The linear part of the force – distance curve was similar comparing the texture analyser and DIC of the gauge section (Fig. 11A and B). From the point where the curve starts to deviate from linearity, the displacement was slower in DIC compared to the texture analyser. This may be explained by slippage between the specimen and the tensile grips, which is not recorded in DIC (Zhou et al., 2013). However, the placement of the virtual extensometer points greatly affected the force – distance curve. When the virtual extensometer was placed around the crack area (Fig. 4B), we observed a much steeper curve compared to the texture analyser and the DIC of the gauge section in both isotropic and anisotropic products (Fig. 11A and B). This was due to the smaller distance when the virtual extensometer is placed around the crack area. Other than that, the ascending branch was relatively unaffected by using DIC. However, the descending branch of the force-distance curve recorded with DIC around the crack exhibited a more gradual decrease compared to both DIC over the gauge length and the texture analyser (Fig. 11A and B). DIC can measure displacement at many points along the surface of the material, providing a higher resolution of the displacement field. Independent of the product (isotropic versus anisotropic), distance recording with DIC enabled us to identify fracture properties that were otherwise not visible.

**Fig. 12:**
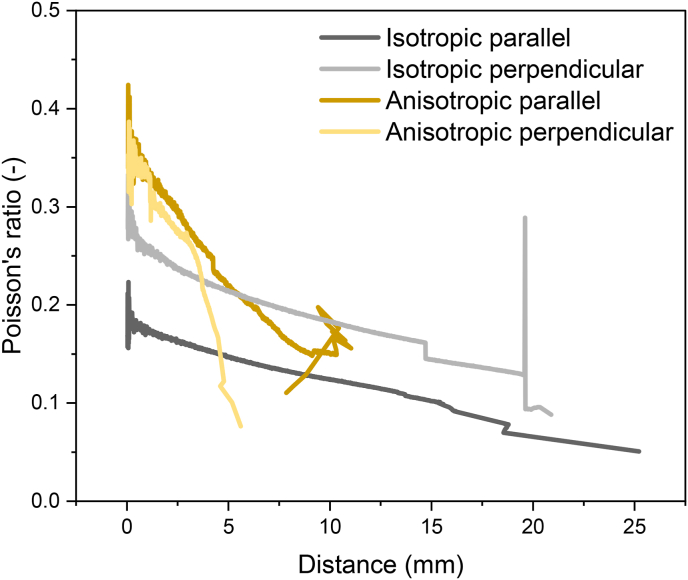
Dynamic Poisson's ratio over distance during tensile testing for isotropic (grey) and anisotropic (yellow) products parallel (dark) and perpendicular (light) to the shear direction.

In calculations of the tensile stress and strain, a constant volume (Poisson's ratio of 0.5) is often assumed (van Vliet, 2013). However, a dynamic Poisson's ratio during tensile testing can be calculated based on DIC-results. The Poisson's ratio offers a consistent means to compare the structural capabilities of actual materials, irrespective of their homogeneity (Greaves et al., 2011). The Poisson's ratio of both isotropic and anisotropic specimen decreased upon uniaxial extension in both the parallel and perpendicular direction to shear (Fig. 12). This behaviour agrees with the nature of inelastic polymer materials (Zhou et al., 2013). The decrease in Poisson's ratio was greater in anisotropic specimen (from 0.43 to 0.11) than in isotropic specimen (from 0.18 to 0.05). Overall, at small deformation, anisotropic specimen had a higher Poisson's ratio than isotropic specimen, indicating that anisotropic samples were relatively elastic. The higher Poisson's ratio of the anisotropic specimen may be attributed to greater porosity in these samples (Jahanbakhshian et al., 2018). As the void spaces increase, there is a corresponding increase in the axial strain compared to the lateral strain. Previous research also showed that air bubbles in meat analogues produced from calcium caseinate played a crucial role in fibrousness (Wang et al., 2019). Upon larger deformation, the Poisson's ratio of anisotropic specimen quickly decreased to values below that of isotropic specimen. This agrees with previous research on rheological properties of isotropic and anisotropic products made from soy, where anisotropic products exhibited a more plastic behaviour compared to a more elastic behaviour in isotropic products (Schreuders et al., 2021b). Furthermore, the Poisson's ratio of both isotropic and anisotropic specimen was dependent on measurement direction (Fig. 12). Even though isotropic products were not sheared, the Poisson's ratio in the perpendicular direction was higher than in parallel direction, suggesting probably less breakdown in this direction. In anisotropic specimen, after an initial period of overlap, the Poisson's ratio decreased faster in the perpendicular direction than in the parallel direction. It is worth noting that the Poisson's ratio was determined over the entire gauge length and width of the tensile specimen, including the crack area. The strain in the tensile direction (y) thus includes both elongation of the material and crack opening. The strain in the other direction (x) only includes elongation of the material.

**Fig. 13:**
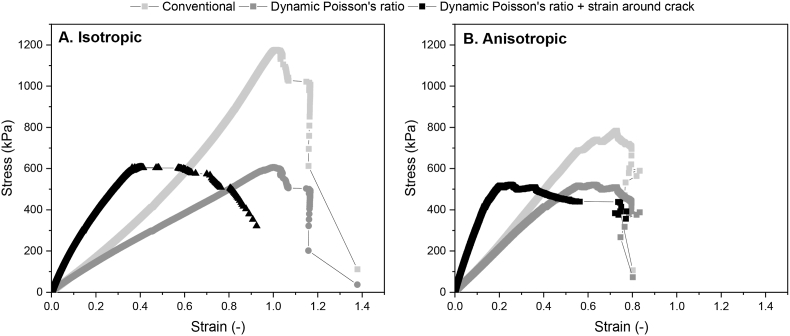
Stress-strain curves of A) isotropic and B) anisotropic products parallel to the direction of shear calculated the conventional way (assumption Poisson's ratio 0.5), with a dynamic Poisson's ratio, and with a dynamic Poisson's ratio and strain calculated around the crack area.

Fig. 12 showed that the assumption of a Poisson's ratio of 0.5 does not hold true for the materials in this study. Thus, the dynamic Poisson's ratio was implemented in calculations of the stress-strain curves as shown in Fig. 13. The contribution of the dynamic Poisson's ratio was accounted for in the calculation of the stress by the cross-sectional area (*A(t)*) with equation (5):

(Eq. 5) $$A\left(t\right)={width}_{DIC}\left(t\right)\times \left(\frac{{width}_{DIC}\left(t\right)}{{width}_{0}}\times {thickness}_{0}\right)$$

where *A(t)* is the cross-sectional area dependent on time, *width*_*DIC*_ *(t)* is the width over time recorded by DIC, *width*_*0*_ is the width at *t* = 0, and *thickness*_*0*_ is the thickness at *t* = 0. Here, we made the assumption that the thickness of the tensile specimen decreased with the same fraction as the width of the specimen. The use of two camera viewpoints would enable direct measurement of the decrease in thickness and, hence, an even more precise determination of the cross-sectional area.

The use of a dynamic Poisson's ratio instead of a static Poisson's ratio (of 0.5) led to a decreased value for the stress in both the isotropic and the anisotropic products (Fig. 13A and B). Moreover, it became clear that in the anisotropic specimen mostly the non-linear part of the graph decreased with a dynamic Poisson's ratio. On the other hand, in the isotropic specimen, stress reduced from the curve's onset when using the dynamic Poisson's ratio. Similar to the force-distance curves (Fig. 11), the placement of the virtual extensometer around the crack area influenced the stress-strain curves as well (Fig. 13). Both the isotropic and anisotropic specimen showed a steeper increase in stress when analysing only the area around the crack. Furthermore, both products also showed a slower descending branch of the curve, facilitating the interpretation of fracturing behaviour. More specifically, the anisotropic specimen clearly showed a lag phase between maximum stress and fracture, where the stress only decreased slightly (Fig. 13B). The stress in the isotropic specimen, on the other hand, decreased gradually after the maximum stress had been reached (Fig. 13A). The placement of the virtual extensometer around the gauge section of the specimen provided a more representative measurement of the overall deformation behaviour of the material. On the other hand, the virtual extensometer placed around the crack provided information about the local deformation and can be useful to understand fracture mechanisms.

### 3.5 The use of DIC analysis to obtain information on fracturing behaviour

**Fig. 14:**
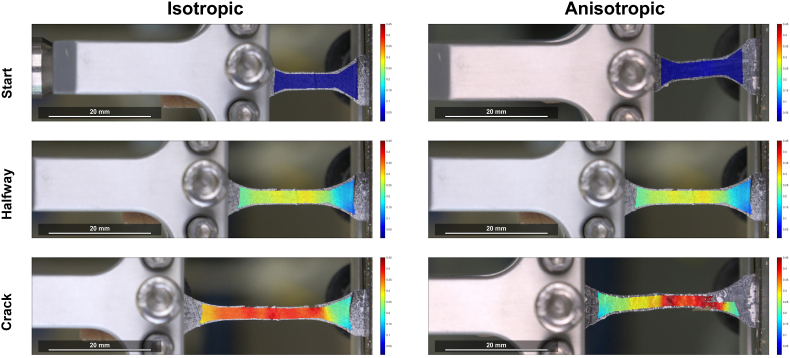
DIC images of start, halfway and just before cracking of tensile test analysis of an isotropic and anisotropic products parallel to the shear flow direction. Images were taken based on the distances defined in Fig. 11. Axis bounds are set between 0.01 and 0.45 strain. Scale bar depicts 20 mm.

The starting, halfway and cracking DIC images of an isotropic and anisotropic specimen are shown in Fig. 14. Regions in blue indicate areas that experienced less deformation, while regions in red indicate areas that experienced more deformation and are, thus, likely areas for fracture to occur. The starting and halfway images of the isotropic and anisotropic specimen were relatively similar, while there were clear differences in the cracking images (Fig. 14). This suggests that differences between isotropic and anisotropic food products mainly occurred in the large deformation area. This observation aligns with the similarity in Young's Modulus and differences in large deformation tensile parameters as previously described (Fig. 8). The crack images revealed that the isotropic specimen had a homogeneous strain distribution, while the anisotropic specimen had a heterogeneous strain distribution. The anisotropic specimen showed highly localized strain, depicting weaker areas in the sample that are more prone to cracking. This can be explained by the large plastic deformation of the anisotropic specimen (Godara et al., 2009), as also observed in section 3.4. When a specific region within the anisotropic specimen undergoes plastic flow, it serves as a focal point for maximal energy absorption or accumulation of damage, thereby safeguarding other regions from experiences substantial damage (Godara et al., 2009). The plastic contribution is due to the addition of a dispersed phase in the anisotropic specimen, while the isotropic specimen were considered a more homogenous matrix consisting of a single continuous phase (Dekkers et al., 2016). The interfaces between the continuous and dispersed phase in the anisotropic products are relatively weak areas that are more prone to cracking. Previous research found that addition of egg white or whey protein to surimi gels led to higher local strain concentrations in tensile tests (Park et al., 2023). They suggested that an increase in particle-particle interactions decreased the water-particle interactions, thereby decreasing the elasticity of the samples.

DIC is computationally intensive, especially when working with large deformations. This is because DIC requires the analysis of large amounts of image data and is, therefore, time consuming. Currently, the use of DIC for large numbers of samples is not feasible, as it would take an unreasonable amount of time and computational resources to process all data. Therefore, DIC is typically used for a limited number of samples. Despite these limitations, DIC turns out to be a valuable tool for characterizing the mechanical properties of food materials, such as anisotropic meat analogues.

## 4 Conclusions

In this study, the effects of tensile gauge length, deformation rate, and digital image correlation on the interpretation of mechanical properties of food products were analysed. We showed that a smaller gauge length and a lower deformation rate led to better visibility of the multi-stage fracture zone, especially for anisotropic products. Toughness was an important parameter to characterize mechanical properties of food, as it showed profound differences between homogeneous, isotropic products and heterogenous, anisotropic products. Furthermore, the true distance data obtained from DIC was found to be more accurate than the distance data obtained from the texture analyser. The placement of the virtual extensometer points around the crack area further increased the accuracy and interpretability of the results in DIC. The use of DIC allowed for a better differentiation between the isotropic and anisotropic food products from a strain distribution perspective. By continuously monitoring the strain in the gauge section of the sample during testing, DIC could be used to maintain a constant strain in the gauge section of the specimen. Overall, this study highlights the importance of careful selection of testing parameters and techniques in evaluating mechanical properties of meat analogues. Therefore, we recommend to use short tensile gauge lengths and DIC for a dynamic Poisson's ratio and true distance recording in tensile testing of anisotropic foods. The placement of a virtual extensometer around the crack area is advised for obtaining information on fracture mechanics. Furthermore, a stable fracturing process and a better measurement of the descending branch can be obtained by controlling the deformation around the crack, as measured by a virtual extensometer. Last, we advise to examine various tensile deformation rates and to demonstrate the differences that emerge. It is important to emphasize that we are not claiming advanced tensile testing only is sufficient to draw conclusions about the fibrous nature of food products. Therefore, future work could involve systematically varying the ratio of pectin to soy protein isolate and coupling this to microstructure characterization to study the relationship between structural attributes and mechanical properties.

## CRediT authorship contribution statement

**Miek Schlangen:** Conceptualization, Investigation, Writing – original draft, Writing – review & editing. **Erik Schlangen:** Conceptualization, Writing – review & editing. **Atze Jan van der Goot:** Conceptualization, Writing – review & editing, Supervision.

## Declaration of competing interest

None.

## Data Availability

Data will be made available on request.
